# Supplementary material for: Theoretical Study on Adsorption Behavior of SF6 Decomposition Components on Mg-MOF-74
Source: Nanomaterials (Basel). 2023 May 23;13(11):1705. doi: 10.3390/nano13111705 (PMC10254539; doi:10.3390/nano13111705)
Supplement: Supplementary file 1 [file nanomaterials-13-01705-s001.zip › nanomaterials-2386643-supplementary.pdf]

Table S1 The sum of Van der waals radius and covalent radius

Gas	Action atom	The sum of Van der Waals radius (Å)	The sum of covalent radius (Å)
SF_6	Mg—F	3.44	2.08
CF_4	Mg—F	3.44	2.08
CS_2	Mg—S	3.87	2.38
H_2S	Mg—S	3.87	2.38
SO_2	Mg—O	3.45	2.09
SO_2F_2	Mg—O	3.45	2.09
SOF_2	Mg—O	3.45	2.09

Table S2 Changes in bond length and bond angle before and after SF_6 molecules adsorption

Bond/angle	Bond length Before adsorption (Å)	Bond length After adsorption (Å)	Variable Quantity (Å)	Bond angel before Adsorption (°)	Bond angel after Adsorption (°)	Variable Quantity (°)
S(1) - F(1)	1.581	1.57391	-0.0071	-	-	-
S(1) - F(2)	1.581	1.57638	-0.0046	-	-	-
S(1) - F(3)	1.581	1.57714	-0.0039	-	-	-
S(1) - F(4)	1.581	1.57928	-0.0017	-	-	-
S(1) - F(5)	1.581	1.61249	0.0315	-	-	-
S(1) - F(6)	1.581	1.57625	-0.0048	-	-	-
F(1)-S(1)-F(2)	-	-	-	90.00	90.1991	0.1991
F(1)-S(1)-F(3)	-	-	-	90.00	90.0799	0.0799
F(1)-S(1)-F(4)	-	-	-	180.00	178.7180	-1.2820
F(1)-S(1)-F(5)	-	-	-	90.00	89.6022	-0.3978
F(1)-S(1)-F(6)	-	-	-	90.00	90.7303	0.7303
F(2)-S(1)-F(3)	-	-	-	180.00	178.8050	-1.1950
F(2)-S(1)-F(4)	-	-	-	90.00	89.8452	-0.1548
F(2)-S(1)-F(5)	-	-	-	90.00	89.9429	-0.0571
F(2)-S(1)-F(6)	-	-	-	90.00	90.5677	0.5677
F(3)-S(1)-F(4)	-	-	-	90.00	89.8499	-0.1501
F(3)-S(1)-F(5)	-	-	-	90.00	88.8971	-1.1029
F(3)-S(1)-F(6)	-	-	-	90.00	90.5906	0.5906
F(4)-S(1)-F(5)	-	-	-	90.00	89.1160	-0.8840
F(4)-S(1)-F(6)	-	-	-	90.00	90.5503	0.5503
F(5)-S(1)-F(6)	-	-	-	180.00	179.3897	-0.6103

Table S3 Changes in bond length and bond angle before and after CF_4 molecules adsorption

Bond/angle	Bond length Before adsorption (Å)	Bond length After adsorption (Å)	Variable Quantity (Å)	Bond angel before Adsorption (°)	Bond angel after Adsorption (°)	Variable Quantity (°)
C(1) - F(1)	1.316	1.34054	0.02454	-	-	-
C(1) - F(2)	1.316	1.30977	-0.00623	-	-	-
C(1) - F(3)	1.316	1.30970	-0.00630	-	-	-

C(1) - F(4)	1.316	1.30800	-0.00800	-	-	-
F(1)-C(1)-F(2)	-	-	-	109.47	109.1049	-0.3651
F(1)-C(1)-F(3)	-	-	-	109.47	108.0986	-1.3714
F(1)-C(1)-F(4)	-	-	-	109.47	108.9980	-0.472
F(2)-C(1)-F(3)	-	-	-	109.47	109.9799	0.5099
F(2)-C(1)-F(3)	-	-	-	109.47	110.4676	0.9976
F(3)-C(1)-F(4)	-	-	-	109.47	110.1428	0.6728

Table S4 Changes in bond length and bond angle before and after CS_2 molecules adsorption

Bond/angle	Bond length Before adsorption (Å)	Bond length After adsorption (Å)	Variable Quantity (Å)	Bond angel before Adsorption (°)	Bond angel after Adsorption (°)	Variable Quantity (°)
C(1) - F(1)	1.5562	1.56695	0.01075	-	-	-
C(1) - F(2)	1.5562	1.54702	-0.00918	-	-	-
F(3)-C(1)-F(4)	-	-	-	180.00	179.8291	-0.1709

Table S5 Changes in bond length and bond angle before and after H_2S molecules adsorption

Bond/angle	Bond length Before adsorption (Å)	Bond length After adsorption (Å)	Variable Quantity (Å)	Bond angel before Adsorption (°)	Bond angel after Adsorption (°)	Variable Quantity (°)
C(1) - F(1)	1.349	1.35179	0.00279	-	-	-
C(1) - F(2)	1.349	1.35168	0.00268	-	-	-
F(3)-C(1)-F(4)	-	-	-	92.12	92.8788	0.7588

Table S6 Changes in bond length and bond angle before and after SO_2 molecules adsorption

Bond/angle	Bond length Before adsorption (Å)	Bond length After adsorption (Å)	Variable Quantity (Å)	Bond angel before Adsorption (°)	Bond angel after Adsorption (°)	Variable Quantity (°)
C(1) - F(1)	1.454	1.47074	0.01674	-	-	-
C(1) - F(2)	1.454	1.45433	0.00033	-	-	-
F(3)-C(1)-F(4)	-	-	-	118.59	116.4651	-2.1249

Table S7 Changes in bond length and bond angle before and after SO_2F_2 molecules adsorption

Bond/angle	Bond length Before adsorption (Å)	Bond length After adsorption (Å)	Variable Quantity (Å)	Bond angel before Adsorption (°)	Bond angel after Adsorption (°)	Variable Quantity (°)
C(1) - F(1)	1.575	1.56815	-0.00685	-	-	-
C(1) - F(2)	1.575	1.56689	-0.00811	-	-	-
C(1) - F(3)	1.424	1.42102	-0.00298	-	-	-
C(1) - F(4)	1.424	1.43450	0.01050	-	-	-
F(1)-C(1)-F(2)	-	-	-	94.8349	95.3059	0.4710

F(1)-C(1)-F(3)	-	-	-	107.9328	108.0827	0.1499
F(1)-C(1)-F(4)	-	-	-	107.9394	105.7348	-2.2046
F(2)-C(1)-F(3)	-	-	-	107.9328	108.6352	0.7024
F(2)-C(1)-F(3)	-	-	-	107.9394	107.7750	-0.1644
F(3)-C(1)-F(4)	-	-	-	125.8551	126.7594	0.9043

Table S8 Changes in bond length and bond angle before and after SOF_2 molecules adsorption

Bond/angle	Bond length Before adsorption (Å)	Bond length After adsorption (Å)	Variable Quantity (Å)	Bond angel before Adsorption (°)	Bond angel after Adsorption (°)	Variable Quantity (°)
C(1) - F(1)	1.616	1.60000	-0.01600	-	-	-
C(1) - F(2)	1.616	1.60589	-0.00589	-	-	-
C(1) - F(3)	1.437	1.44700	0.01000	-	-	-
F(2)-C(1)-F(3)	-	-	-	92.64	93.6346	0.9946
F(2)-C(1)-F(3)	-	-	-	106.71	106.7621	0.0521
F(3)-C(1)-F(4)	-	-	-	106.71	106.6037	-0.1063
